# Supplementary material for: The interactome of intact mitochondria by cross-linking mass spectrometry provides evidence for coexisting respiratory supercomplexes
Source: Mol Cell Proteomics. 2017 Dec 8;17(2):216–32. doi: 10.1074/mcp.RA117.000470 (PMC5795388; doi:10.1074/mcp.RA117.000470)
Supplement: Supplemental Data [file supp_RA117.000470_133922_0_supp_23978_fzffwf.zip › spectra_annotation/mito_spectra_annotation/41-1-6-1-4-1.pdf]

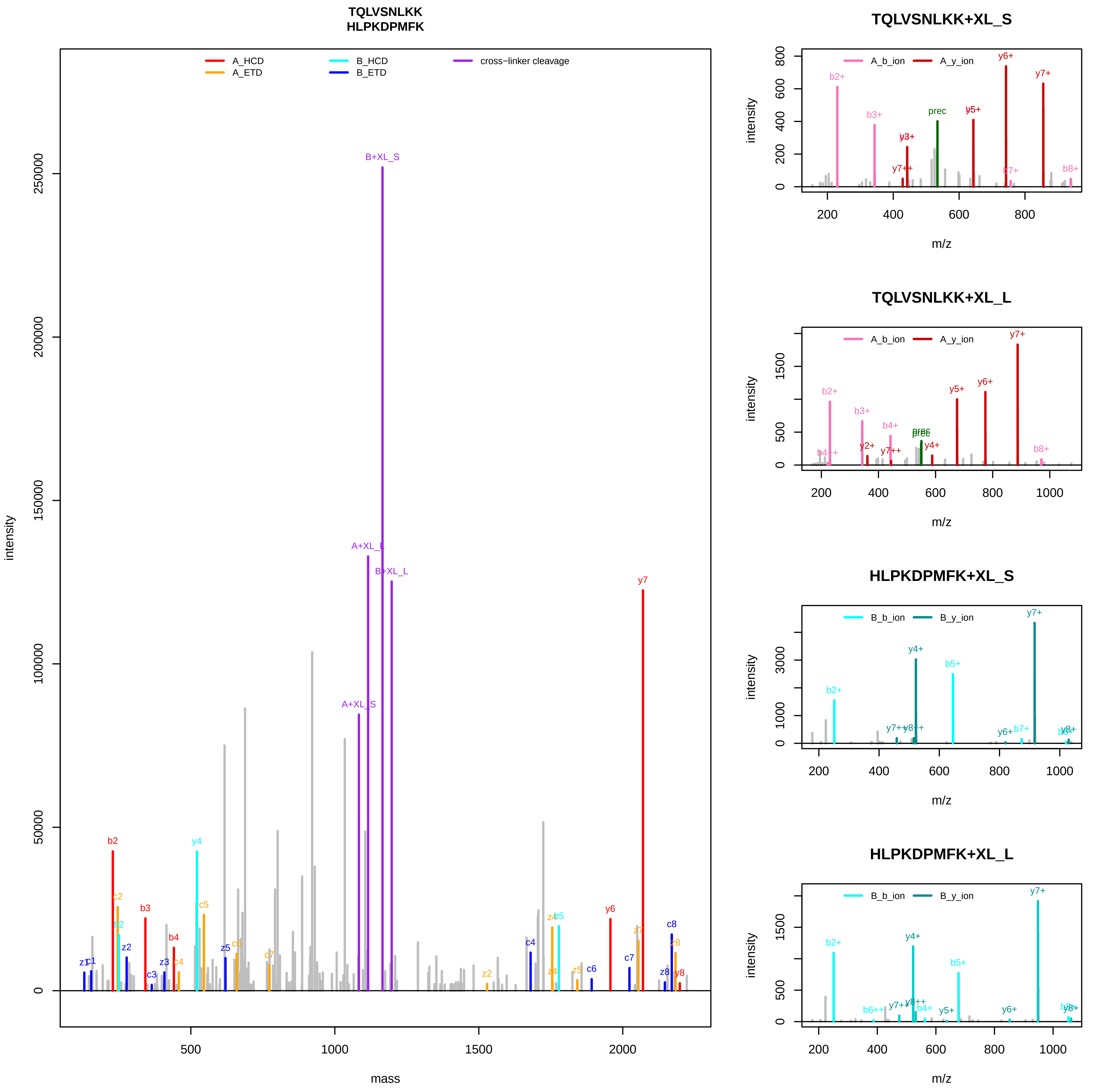
TQLVSNLKK
HLPKDPMFK
A_HCD
A_ETD
B_HCD
B_ETD
cross−linker cleavage
B+XL_S
A+XL_L
B+XL_L
A+XL_S
y7
b2
y4
b3
c2
c5
b4
c4
z5
c6
c7
y6
c8
z8
y8
c4
b5
z4
z5
c6
c7
z2
intensity
mass
0
50000
100000
150000
200000
250000
500
1000
1500
2000
TQLVSNLKK+XL_S
A_b_ion
A_y_ion
b2+
b3+
y3+
prec
y5+
y6+
y7+
y7++
b7+
b8+
TQLVSNLKK+XL_L
b2+
b3+
b4+
y2+
y7++
prec
y4+
y5+
y6+
y7+
b8+
HLPKDPMFK+XL_S
B_b_ion
B_y_ion
b2+
y7++
y8++
y4+
b5+
y6+
b7+
y7+
y8+
HLPKDPMFK+XL_L
b2+
b6++
y7++
y8++
y4+
b4+
y5+
b5+
y6+
y7+
y8+
intensity
m/z
